# NanoPASS: an easy-to-use user interface for nanoparticle dosimetry with the 3DSDD model

**DOI:** 10.1186/s12989-020-00368-w

**Published:** 2020-09-18

**Authors:** Falko Frenzel, Laura König-Mattern, Valerie Stock, Linn Voss, Maxi B. Paul, Holger Sieg, Albert Braeuning, Andreas Voigt, Linda Böhmert

**Affiliations:** 1 German Federal Institute for Risk Assessment, Department of Food Safety, Max-Dohrn-Str. 8-10, 10589 Berlin, Germany; 2 Otto-von-Guericke University Magdeburg, Chair of Process Systems Engineering, Universitätsplatz 2, 39016 Magdeburg, Germany

**Keywords:** Dosimetric calculation, Nanotoxicology, In vitro, Cell culture, Caco-2, HepaRG

## Abstract

Nanoparticles exhibit a specific diffusion and sedimentation behavior under cell culture conditions as used in nantoxicological in vitro testing. How a particular particle suspension behaves depends on the particular physicochemical characteristics of the particles and the cell culture system. Only a fraction of the nanoparticles applied to a cell culture will thus reach the cells within a given time frame. Therefore, dosimetric calculations are essential not only to determine the exact fraction of nanoparticles that has come into contact with the cells, but also to ensure experimental comparability and correct interpretation of results, respectively. Yet, the use of published dosimetry models is limited. Not the least because the correct application of these in silico tools usually requires bioinformatics knowledge, which often is perceived a hurdle. Moreover, not all models are freely available and accessible. In order to overcome this obstacle, we have now developed an easy-to-use interface for our recently published 3DSDD dosimetry model, called NanoPASS (NanoParticle Administration Sedimentation Simulator). The interface is freely available to all researchers. It will facilitate the use of in silico dosimetry in nanotoxicology and thus improve interpretation and comparability of in vitro results in the field.

## Introduction

The number of in vitro studies on particle uptake, effects and toxicity is increasing. While the main focus of toxicological research has been in the field of inorganic nanoparticles, plastic particles, especially microplastics, have recently also come into focus [2]. Small-sized particles differ by size, shape, surface coating and agglomeration behavior, and show a particular behavior with respect to their concentration distribution in dispersion: within a given time frame, only a fraction of the applied particles will come into contact with cells located at the bottom of a cell culture vessel [10, 11]. This fraction, termed “effective dose”, “delivered dose” or “target cell dose” depends on the physicochemical characteristics of the particles and of the liquid, e.g. the cell culture medium. Together, these characteristics determine how sedimentation and diffusion affect particle distribution over time [3, 12, 13]. Dosimetry, i.e. the determination of the effective dose of particles that have come into contact with cells in in vitro experiments, is a major issue in the field of particle toxicology and a prerequisite for comparability and correct interpretation of results [7, 13]. A number of models and approaches for dosimetry calculations in silico have been published [4, 6, 8, 9, 14]. Latest result show that that the dose perceived by the cells on the bottom of the well after 24 h of exposure is around 85% lower than the administered nominal media concentration [10]. Usage of these models, however, remains limited. Reasons are the limited public availability of some of the models but also real or perceived hurdles regarding the bioinformatics expertise required for operating the respective software packages and algorithms.

## Aim

Therefore, we aimed to develop an easy-to-use user interface (termed NanoPASS – NanoParticle Administration Sedimentation Simulator) for our 3DSDD (3D-sedimentation-diffusion-dosimetry) model that was published recently [1]. The 3DSDD model was designed with respect to the complex growth behavior of differentiated cell models such as Caco-2 or HepaRG. Such cell models need to be cultivated for differentiation for up to several weeks prior to incubation with the particles. The 3DSDD model calculates the effective dose received by the cells, taking into account different cell populations at the bottom and the lateral walls of the culture dish, the latter resulting from continuous growth of cells after having reached confluency at the bottom. Moreover, as basis of the in silico calculations, the 3DSDD model allows using either the hydrodynamic particle diameter as measured by light scattering methods, or the diffusion coefficient as measured by nanoparticle tracking analysis, offering more flexibility than previous dosimetry models [1]. The NanoPASS user interface will enable all users in the field to calculate the effective dose of their specific particles in silico, particularly for subsequent use in vitro. The interface works with the freely available software R and RStudio and can be used without knowledge of programming in R. Moreover, we present a detailed manual how to install and use 3DSDD via NanoPASS. Thereby NanoPASS helps to better implement dosimetry in nanotoxicology and therefore facilitates interpretation and comparison of results obtained with different in vitro models and particles.

## How to use the user interface NanoPASS

Both the 3DSDD model and the NanoPASS user interface are freely available for use in research. When using NanoPASS, the user can enter all the parameters into the entry mask of the user interface and is presented with an overview of the results expressed in different graphics.

**Fig. 1:**
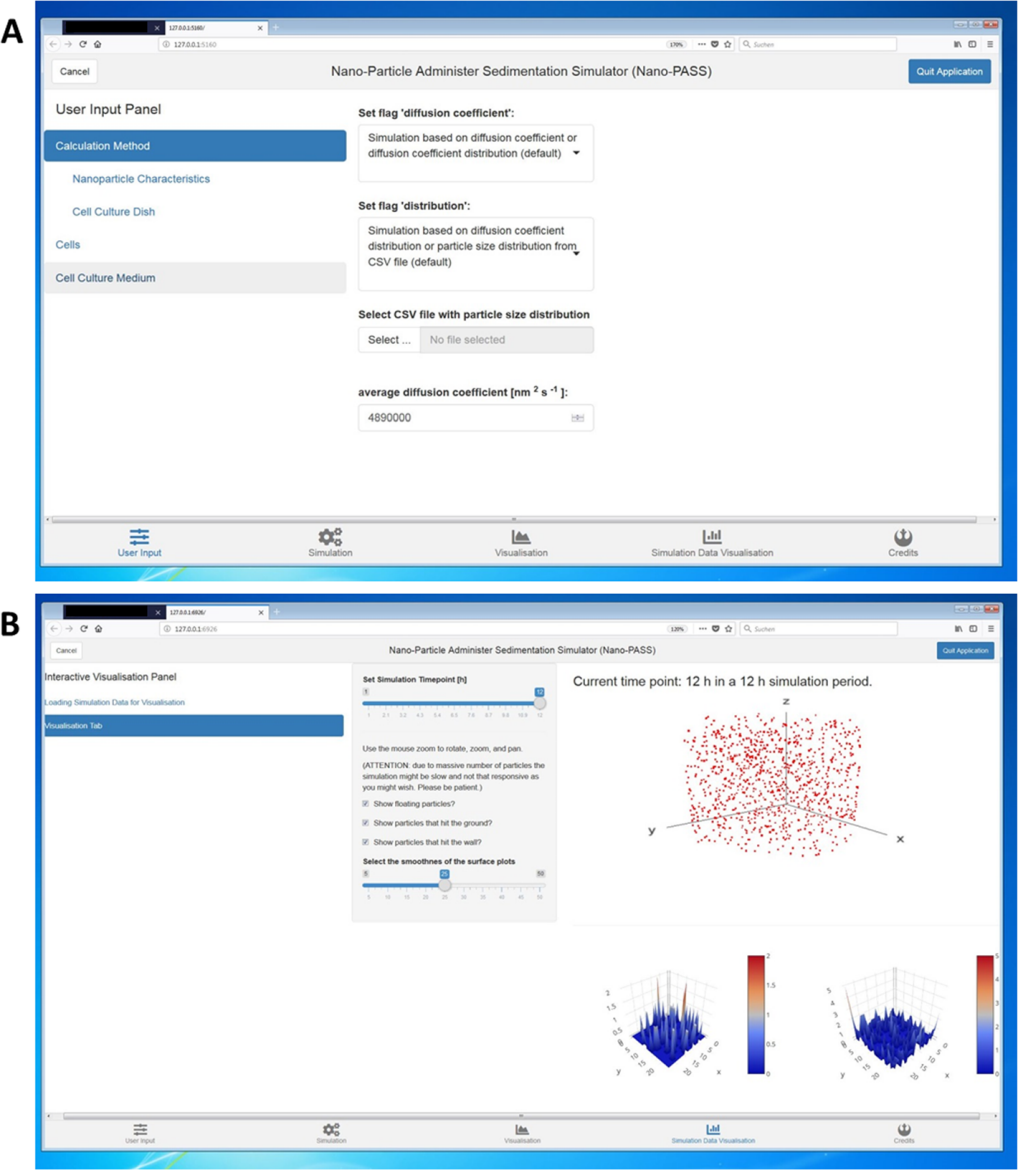
Visual appearance of the dosimetry user interface NanoPASS for calculations with the 3DSDD model. Representative images show the entry mask (**a**) and the results delivered by the software (**b**)

1. We aimed to give as much help and guidance as possible in order to help readers with their effective nanoparticle dose calculations. When using the NanoPASS interface please proceed as follows:
2. Download the NanoPASS installation guide from the supplementary information (Additional file 1).
3. Follow the instructions to install R (v3.6 or later) and RStudio (v1.2 or later).
4. Download the NanoPASS file from GitHub (https://github.com/falfren/NanoPASS) or Additional file 2.
5. Continue to follow the instructions to install the 3DSDD user interface NanoPASS.
6. Open the interface in your browser. Figure 1a gives an impression of the visual appearance of the user interface. A representative image of the output delivered by NanoPASS is presented in Fig. 1b.
7. The interface is separated into 4 tabs shown at the bottom (“User Input”, “Simulation”, “Visualisation”, “Simulation Data Visualisation” and “Credits”). Use the first tab “User Input” to fill in all necessary information about your particles and the in vitro system (for more details about the necessary information see next section).
8. Go to the next tab “simulation“: Check if your data are listed correctly and chose a memory location.
9. Let the system calculate your particle distribution results choosing “start simulation”.
10. Get an impression of your results in the “Simulation Data Visualisation” tap.
11. Choose the desired simulation time point, particles that hit the ground / the wall should be included and export graphs in .png.

## Necessary information for dosimetry calculations

Knowledge of a number of parameters of the nanoparticles, the cell culture dish, and the cell culture medium is necessary to calculate the effective particle dose with the 3DSDD model and the NanoPASS user interface. These parameters can be experimentally determined or estimated. For an overview see Table 1 and the following text or refer to our recent publication [1]. The parameters that are needed refer to different parts of the system that is modeled, namely the characteristics of the nanoparticles, the dimensions of the cell culture dish used for the experiment, characteristics of the cells, characteristics of the cell culture medium and parameters for the calculation model itself.

**Table 1:** Parameters needed for dosimetric calculations with the 3DSDD model

parameter	unit	commonly used methods	comments
**Nanoparticle characteristics**			
hydrodynamic diameter **or** diffusion coefficient	[nm] **or** [nm^2^ s^− 1^]	NTA, DLS **or** NTA	One of the two parameters is needed, either as average or as whole distribution.
effective density	[g cm^− 3^]	volumetric centrifugation method	The effective density of particles is the density of particle agglomerates that are formed for most particles in cell culture medium. For more detail please refer to [3].
**Cell culture dish**			
dish bottom area	[cm^2^]	length measured or manufacturer’s information	
medium filling level	[cm]	height measured or calculated	
**Cell system**			
height of cell growth at the walls	[cm]	height measured	Differentiated cell models like Caco-2 or HepaRG cells push their monolayer up the lateral wall of the culture dish during differentiation (see [5]). For undifferentiated cell models choose 0.
**Cell culture medium**			
medium density	[g cm^−3^]	densitometer	
medium viscosity	[mPa s]	viscometer	
temperature during incubation	[°C]	thermometer	
temperature during particle characterization	[°C]	thermometer	
medium viscosity during particles characterization	[mPa s]	NTA	
**Calculation parameters**			
number of particles simulated	–	–	Selecting more particles yields more detailed results, but increases the computational power needed (recommendation: 10,000 particles).
simulation time	[h]	–	Corresponds to the maximal incubation time of interest.
fraction of an hour a data snapshot is taken	[h]	–	These snapshots are needed for the interactive 3D-representation of the sedimentation process. This parameter defines the time that passes between two steps in the simulation process.

Abbreviations: *NTA* Nanoparticle Tracing Analysis, *DLS* Dynamic Light Scattering

### Nanoparticle characteristics

3DSDD calculates nanoparticles as spherical particles based on size distribution. Prerequisite thus is the determination of the hydrodynamic size in the respective cell culture medium. Please note that measuring in other fluids than the one used for cell incubation may influence the results as the composition of the dispersion medium has an influence on dispersion stability, particle aggregation, ion release and hydrodynamic diameter, respectively. Also, the software will relate to the diameter, not the radius.

When using NTA (Nanoparticle Tracing Analysis) to determine the size of the particles, one can use the diffusion coefficient instead of the hydrodynamic diameter size distribution. As NTA tracks single particles and calculates the hydrodynamic diameter via the diffusion coefficient this actually omits the conversion of the hydrodynamic diameter back into the diffusion coefficient otherwise performed by 3DSDD. An example for particle distribution data is included within the R-package and is further described in the package documentation.

If one does not have the possibility to measure a whole distribution of the particle size or diffusion coefficient, it is also possible to use an average value for the calculation. This will, however, reduce the accuracy of the in silico results.

In addition to the particle size, the software will also require input of the particle density. This is due to the fact that most particles will form agglomerates in cell culture medium and the density allows compensating for any medium inclusions thereof. The effective density therefore needs to be determined in the cell culture medium used for incubation. Merely relying on the material would in most cases cause a massive overestimation of particle sedimentation. Determination of agglomerate density can, for example, be done by analytical centrifugation. Although not common in many toxicological labs, the effective density could also be experimentally estimated by using the PCV tube centrifugation [3]. However, it should be kept in mind that this will provide an average value for the density of the particle agglomerate distribution.

For buoyant particles that have a smaller density then the surrounding fluid and therefore do not sediment to the bottom but float to the surface, the model automatically calculates the floating instead of the sedimentation when the effective density of the particles is smaller than the density of the cell culture medium.

### Cell culture dish

To give a correct 3D calculation of the nanoparticle distribution during incubation, the used format of the incubation well and height of medium fluid column needs to be measured. This can be done by using the information provided by the manufacturer, or simply by measuring the size of the cavities of the incubation plate. It has to be kept in mind that the size of cell culture dishes may vary between manufacturers. Medium fluid column height can also be measured or calculated from the well size and medium volume. Table 2 shows some examples.

**Table 2:**
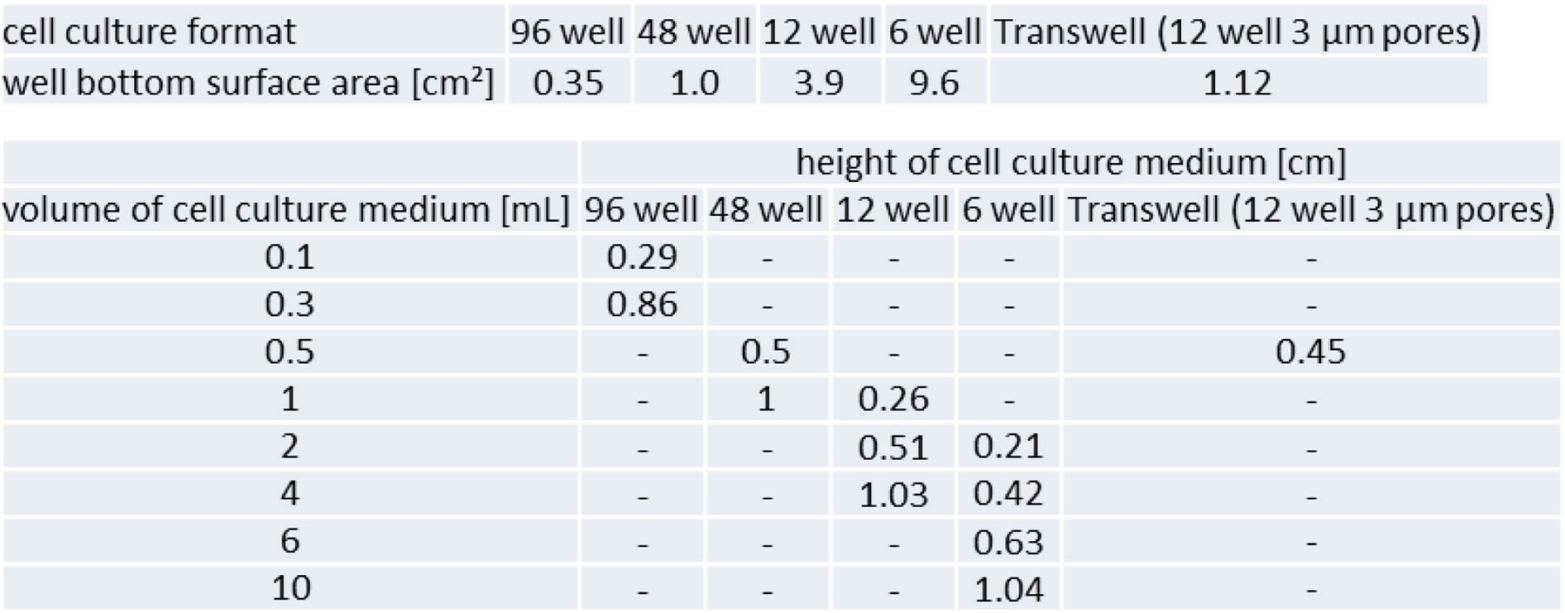
Examples surface areas of the cell culture dish well bottoms and calculated medium fluid column heights (Attention: manufacturer dependent)

### Cell system

Most adherent cell culture systems are incubated for 24 h post seeding, so that the cells have time to attach to the bottom of the cell culture dish. In these cases the dish bottom area is the area of interest. This parameter was already needed to determine the dimensions of the cell culture dish. Please note there some cell culture models might need longer cultivation times prior to incubation in order to allow for cell differentiation. This is the case, for example, for the most common cell model of the intestinal barrier, Caco-2, as well as for the hepatic cell line HepaRG. After reaching confluency Caco-2 cells need up to 21 days of differentiation to express typical intestinal properties such as the microvilli brush border or tight junctions. The respective monolayer typically reaches a height of about 0.54 cm at the walls of the culture dish. Contrastingly HepaRG reach approximately only half of that (about 0.28 cm) following 4 weeks of differentiation. The cell fraction at the walls is of interest especially for nanoparticle incubation, because these cells come in contact with a different amount of particles compared to the fraction at the bottom. Especially in the commonly used 96-well plate format the number of Caco-2 cells at the wall is much greater than that of cells on the bottom.

### Cell culture medium

Likewise some parameters of the incubation medium are needed also for modeling. These include the viscosity and density at the temperature during particle exposure as well as during particle incubation. Especially the density and viscosity will differ slightly with changing medium composition or depending on the additives used. Table 3 provides a few examples.

**Table 3:**
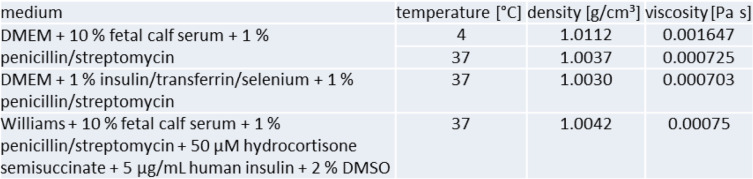
Examples for density and viscosity of different cell culture media at different temperatures

Abbreviations: *DMEM* Dulbecco’s Modified Eagle’s Medium, *DMSO* Dimethyl sulfoxide

### Calculation parameters

Eventually, three parameters for the calculation need to be defined, namely the number of particles simulated, simulation time, and the sampling interval when snapshots are taken. These parameters can be varied. The more particles are simulated the more detailed are the results, but the more computational resources are needed. A number of 10,000 particles is a good compromise as shown in our previous publication [1]. The simulation time should fit your incubation time on your in vitro experiment. Finally, the sampling interval determines how often within an hour the current position of the simulated particles shall be recorded in the save file. These data shall then be used to calculate the interactive graphical interpretation of the three dimensional in silico particle distribution. A value of 0.25 indicates sampling every 15 min over simulation period defined. However, the smaller the fraction the more snapshots will be taken and the graphical representation will be more fluent. As a downside, the resulting file will increase in size, which may cause memory issues on less powerful machines.

## Supplementary information

**Additional file 1.**

**Additional file 2.**

## Data Availability

The in silico model 3DSDD can be found in our previous publication [1]. The user interface NanoPASS presented in this short report can be found in the supplementary information.

## Abbreviations

3DSDD: 3D Sedimentation Diffusion Dosimetry
DLS: Dynamic Light Scattering
DMEM: Dulbecco’s Modified Eagle’s Medium
DMSO: Dimethylsulfoxid
NanoPASS: NanoParticle Administration Sedimentation Simulator
NTA: Nanoparticle Tracing Analysis
