# Supplementary material for: NanoPASS: an easy-to-use user interface for nanoparticle dosimetry with the 3DSDD model
Source: Part Fibre Toxicol. 2020 Sep 18;17:45. doi: 10.1186/s12989-020-00368-w (PMC7502021; doi:10.1186/s12989-020-00368-w)
Supplement: Supplementary file 1 — Additional file 1. [file 12989_2020_368_MOESM1_ESM.pdf]

Supporting Information

Short report

NanoPASS: an easy-to-use user interface for nanoparticle dosimetry with the 3DSDD model

--- NanoPASS installation guide ---

Falko Frenzel[1], Laura König-Mattern[2], Valerie Stock[1], Linn Voss[1], Maxi B Paul[1], Holger Sieg[1], Albert Braeuning[1], Andreas Voigt[2], Linda Böhmert[1,*]

[1] German Federal Institute for Risk Assessment, Department of Food Safety, Max-Dohrn-Str. 8-10, 10589 Berlin, Germany

[2] Otto-von-Guericke University Magdeburg, Chair of Process Systems Engineering, Universitätsplatz 2, 39016 Magdeburg, Germany

Install R and RStudio:

- first install R on your computer (information on how this works for your operating system can be found under https://cran.r-project.org/)
- after installing R, download and install RStudio (https://www.rstudio.com/)
- open RStudio (icon for RStudio should be on your desktop when using recommended installation method)

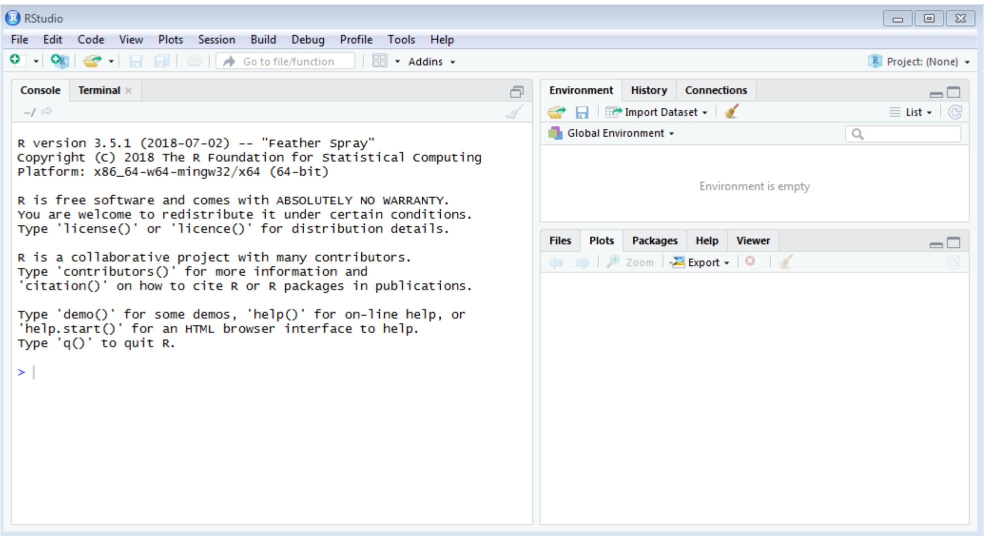

- then you should find the following panel in your RStudio IDE:
  - top right side: global environment (here stored R objects like variables and dataframes are shown);
  - (optional) top left side: script (here you can create a draft of your code);
  - bottom left side: console (here you can type your code directly);
  - bottom right side: (here you can install packages and show plots)

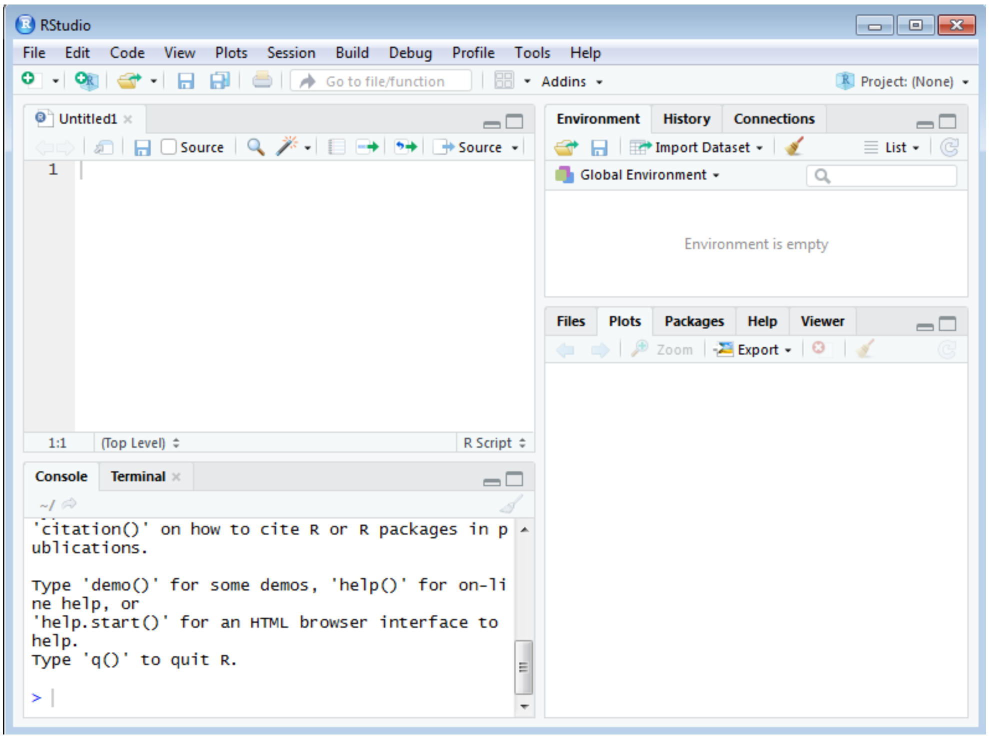

Install 3DSDD user interface NanoPASS:

- install NanoPASS: download NanoPASS from GitHub (https://github.com/falfren/NanoPASS) or the additional file 2 of the paper to your computer
- in RStudio: click on "Packages" and then on "Install" (marked in yellow)

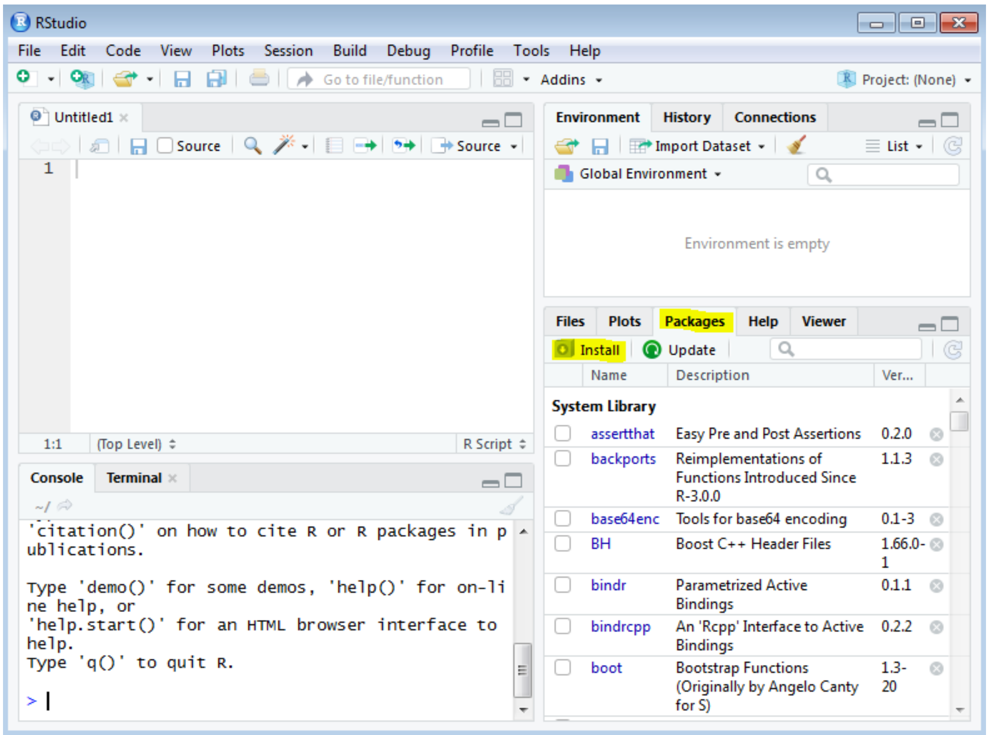

- an additional window opens; change at "Install from" to "Package Archive File", browse for the NanoPASS-File on your computer and select the recently downloaded package file; click "install"

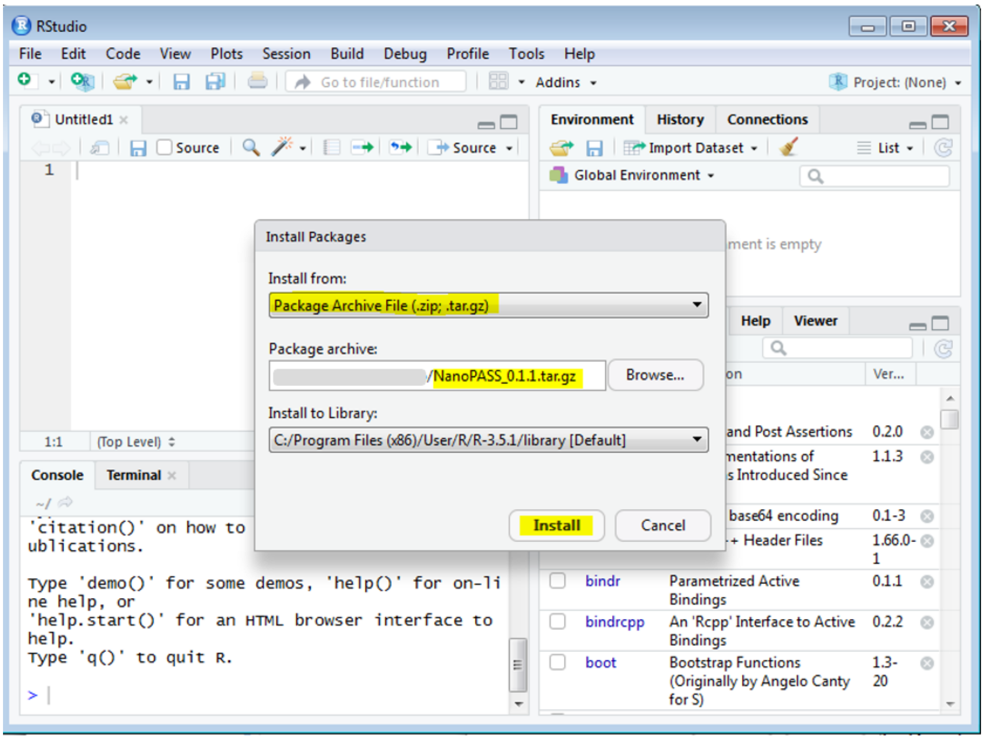

➢ there should be no error in the console.

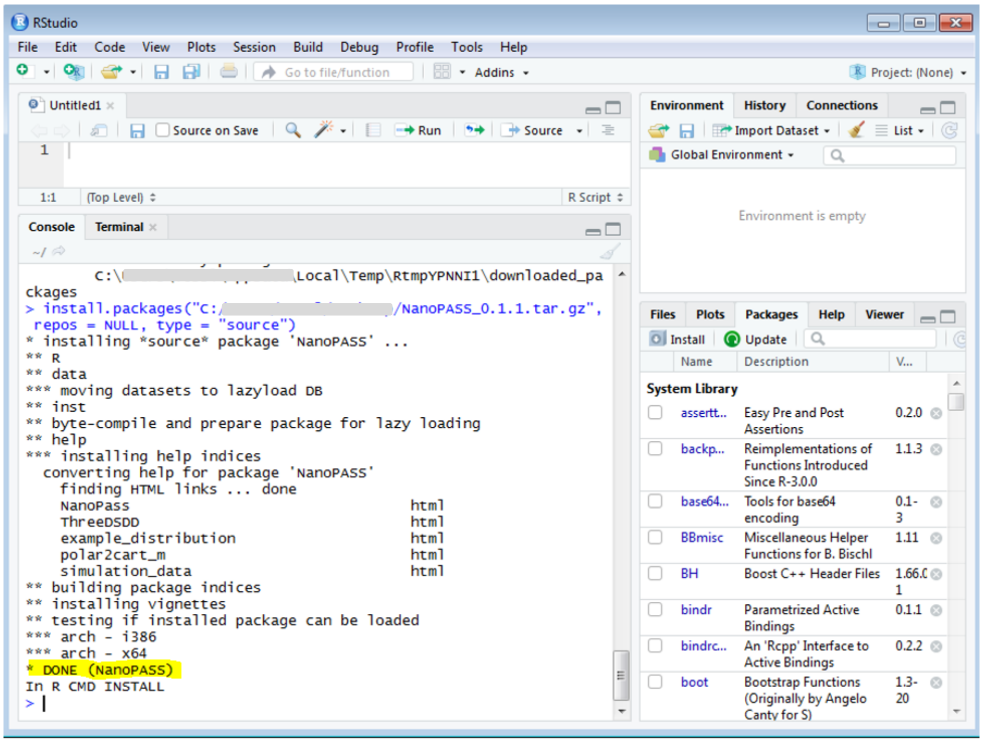

Starting with R 4.0.0 (released April 2020), R for Windows uses a new toolchain bundle for compiling R-packages

- if this additional installation is needed, following error occours:

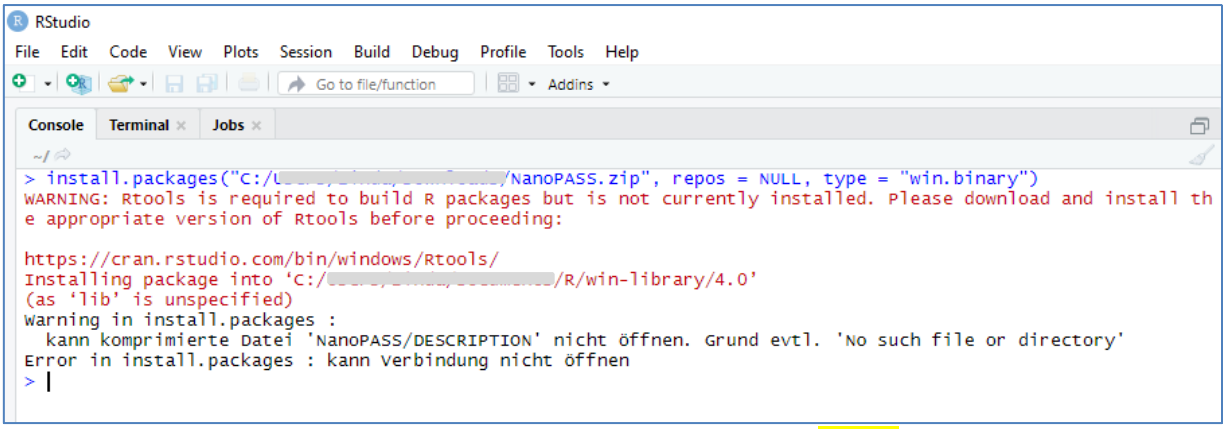

- then you need to follow the instructions marked in yellow and on https://cran.r-project.org/bin/windows/Rtools/

- despite the NanoPASS package setting that all dependencies should be installed during the installation process: if an error occurs because of missing dependencies packages are not installed (as shown in the following picture in yellow), go again to Packages → Install

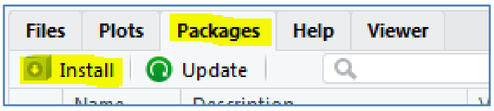

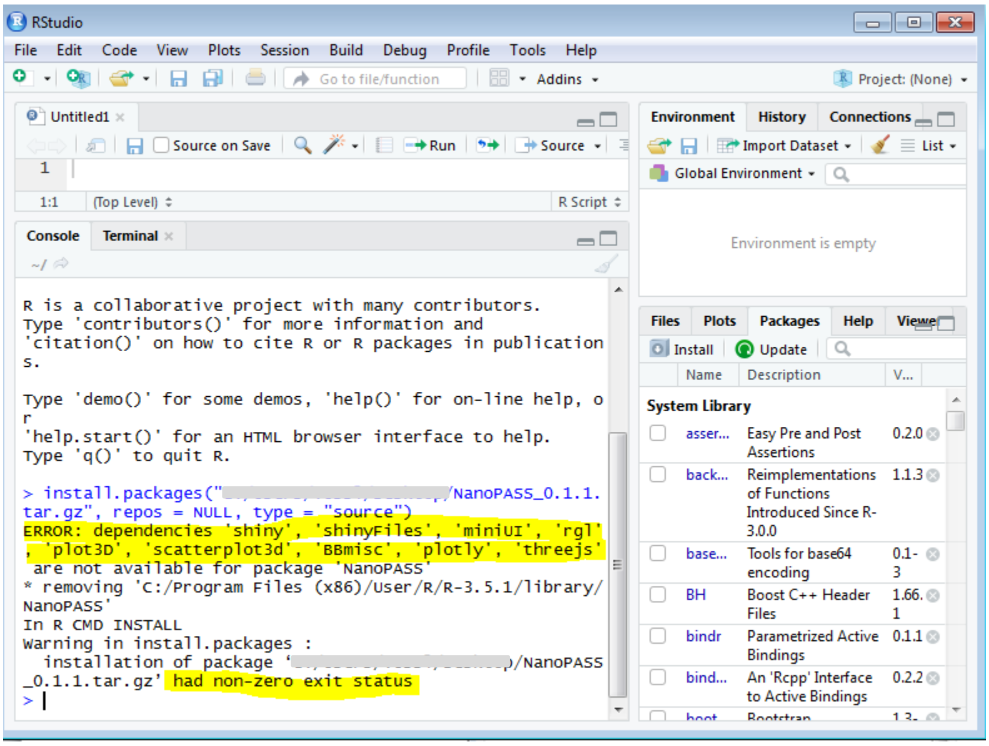

- change at "Install from" to "Repository (CRAN)"; enter missing packages (shown in console in ' ') in the packages search bar (separated by ","); make sure to check the option "Install dependencies" and click on the "Install" button

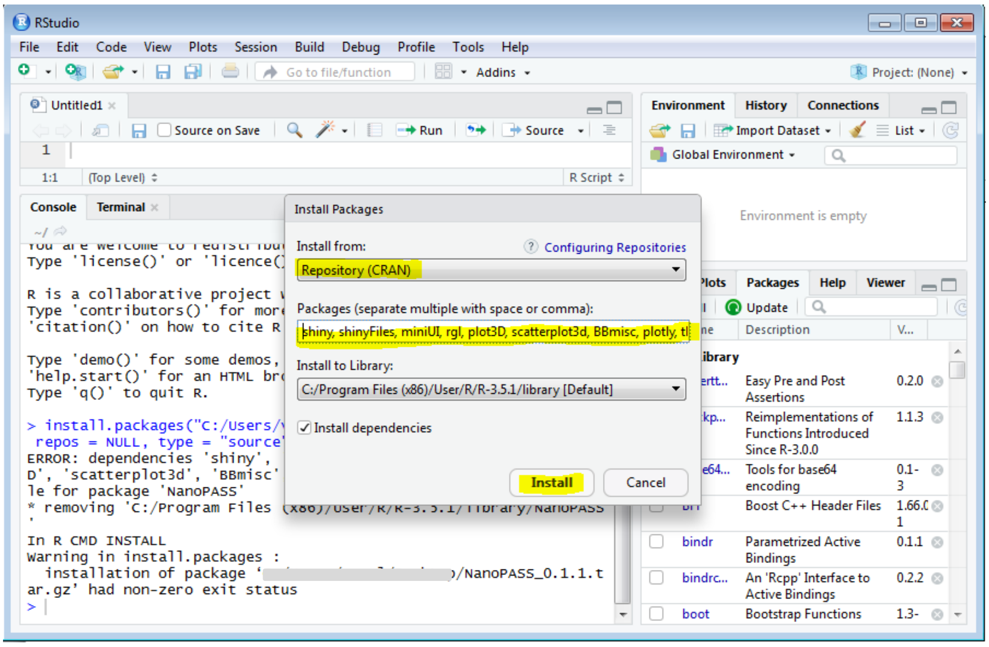

➢ then repeat the installation of NanoPASS

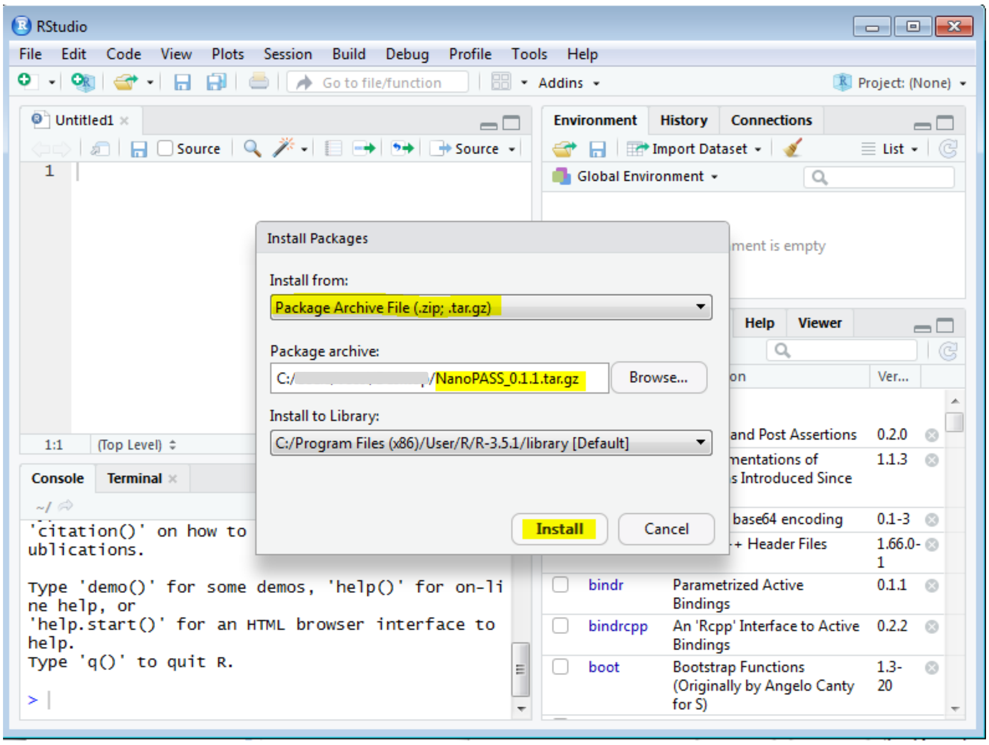

➢ from now on, NanoPASS is installed in R and you do not have to install it again when opening RStudio the next time; you can just start at the next slide

➢ in order to check wether the installation process was successful, NanoPASS shall be listed in the panel “Packages” in the bottom right panel

starting NanoPASS:

➢ open RStudio, enter “NanoPASS::ThreeDSDD()” in the console , Click ENTER; NanoPASS will open in your browser

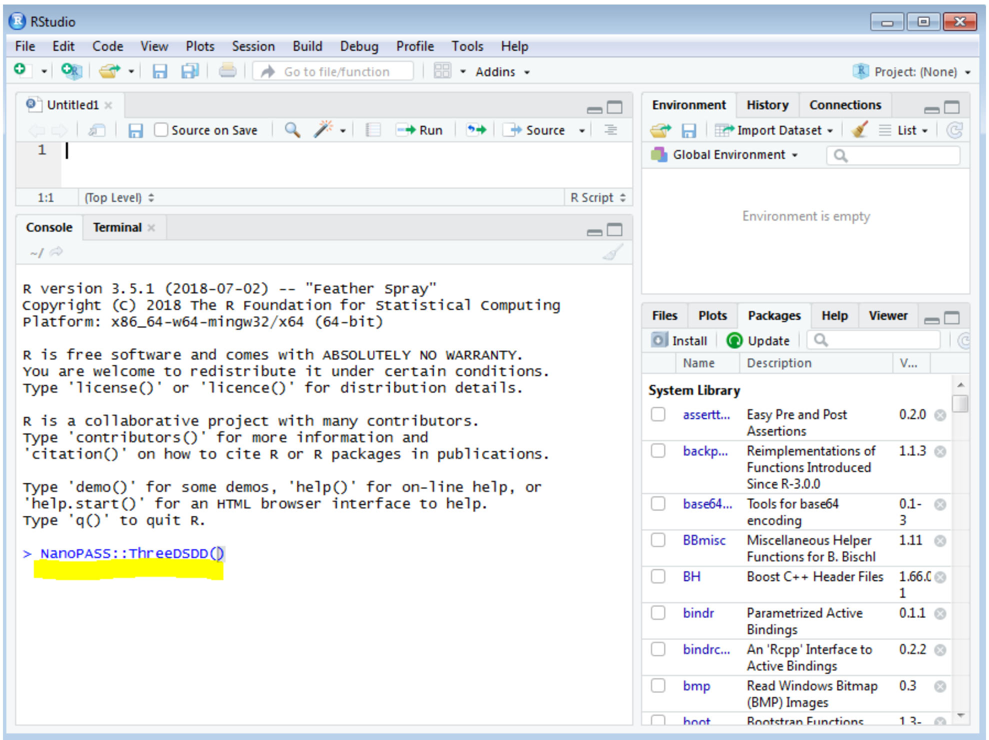

➢ in your web browser, you should see this:

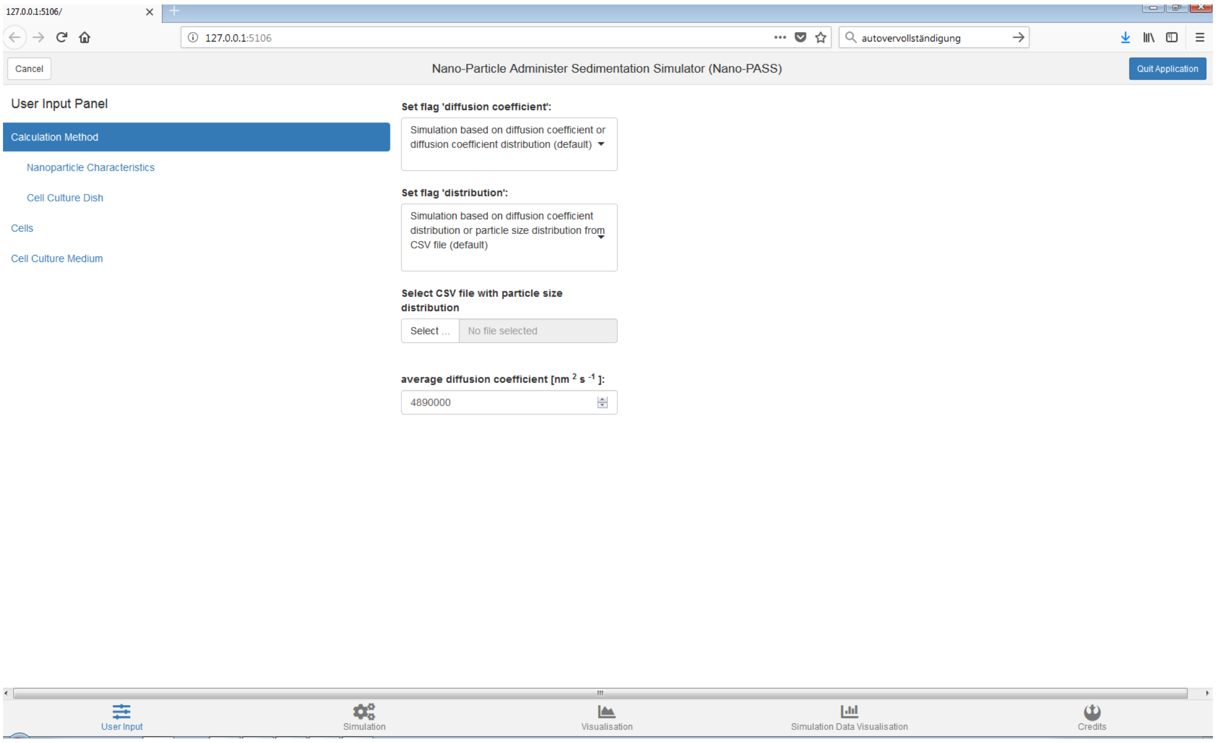

➢ if not, go back to your R-window, here you can find the link as well. Copy that in your browser and you will get to NanoPASS

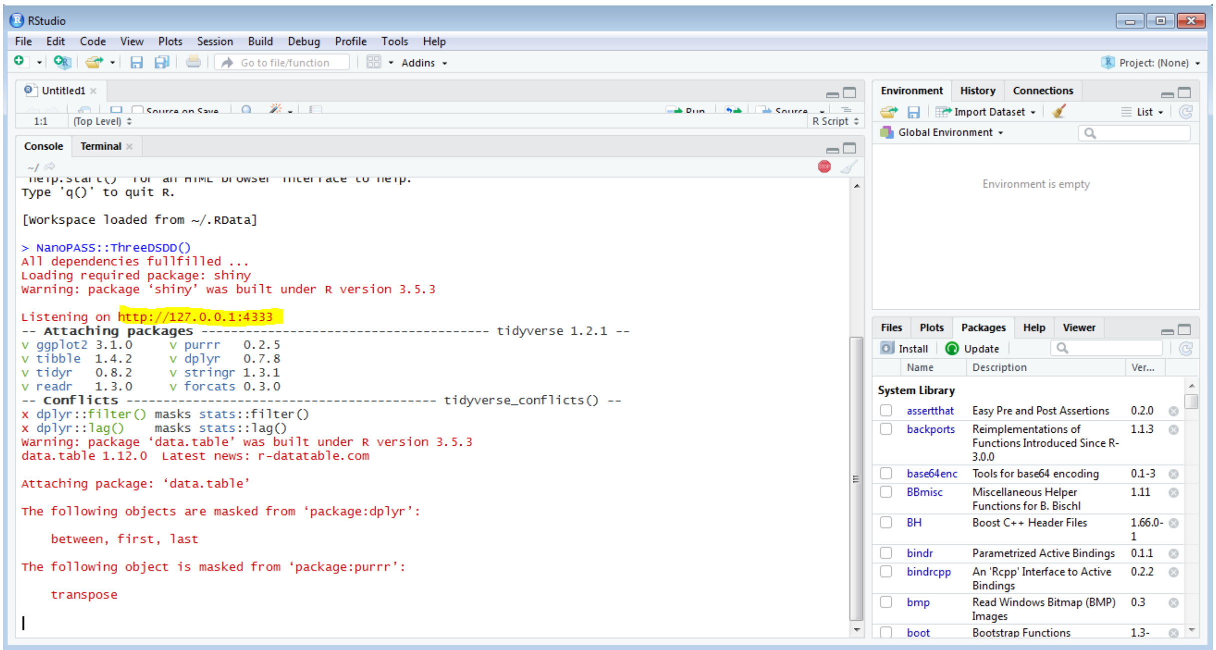

➢ now you can start to use 3DSDD model with the NanoPASS user interface

To quit NanoPASS:

➢ when you are done in your browser, go back to RStudio and Stopp the command by clicking on “Stop“; the “>“ appears, you can now go on
